# Calibrated Ecosystem Models Cannot Predict the Consequences of Conservation Management Decisions—Clarification

**DOI:** 10.1111/ele.70175

**Published:** 2025-10-26

**Authors:** Larissa Lubiana Botelho, Cailan Jeynes‐Smith, Sarah A. Vollert, Michael Bode

**Affiliations:** ^1^ School of Mathematical Sciences Queensland University of Technology Brisbane Queensland Australia; ^2^ Securing Antarctica's Environmental Future, School of Mathematical Sciences Queensland University of Technology Brisbane Queensland Australia; ^3^ Department of Paediatrics The University of Tennessee Health Science Centre Memphis Tennessee USA; ^4^ Centre for Data Science Queensland University of Technology Brisbane Queensland Australia

**Keywords:** decision support, ecological forecasting, ecosystem modelling, microcosms, uncertainty analysis

## Abstract

Woodstock and Harris raise concerns about generalisations in our paper ‘Calibrated Ecosystem Models Cannot Predict the Consequences of Conservation Management Decisions’. They implied that our conclusions are invalidated by the exclusion of density dependent compensatory feedback and the existence of automated calibration approaches with overfitting penalties—claims that overlook the broader structural issues we identified. Here, we explore their key criticism in more detail, clarify our position and explain why we continue to stand by our findings.

## Graphical abstract

Well‐calibrated ecosystem models can produce divergent predictions despite similar parameter sets—and similar predictions despite dissimilar ones—due to hidden issues such as parameter non‐identifiability, model misspecification, numerical instability, and the curse of dimensionality. These problems compromise not only predictive power but also our ability to reliably describe underlying system dynamics, underscoring the need for rigorous model validation.

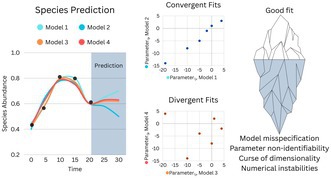

1

In our original study (Lubiana Botelho 2025), we focused on a specific class of ecosystem models: those that aim to describe or predict the abundance or biomass of multiple interacting species. These models are central to many conservation applications, especially for aiding decision‐making for threatened or managed species (Baker 2019; Peterson 2021). We recognise that the term ecosystem models encompass a wide range of approaches, including biogeochemical models (which often do not represent individual species explicitly, e.g., (Daewel 2019)) or single species models that incorporate environmental factors, e.g., (Stock 2021).

Our paper did not—and could not—claim that all ecosystem models are inherently unreliable. Rather, we showed that well‐calibrated multi‐species ecosystem models can still fail to predict intervention outcomes. While a single study cannot determine how widespread this issue is, the existence of such failures, despite strong calibration and ideal data, raises important concerns about the assumptions underlying conservation decision‐making based on model projections.

Despite Woodstock and Harris's (Woodstock 2025) criticism that simple Lotka‐Volterra models cannot simulate realistic predator–prey dynamics, these models remain extensively used (Fronhofer 2015; Geary 2020; Liao 2020; Maynard 2020; Sahasrabudhe 2011). Moreover, the problem was not that these models failed to fit the ecosystem dynamics—they produced thousands of parameter combinations yielding similarly good fits across hundreds of microcosms. The problem is that this apparent success is deceptive: although the models fit the data equally well, they disagreed on species interactions and failed to reliably predict future dynamics or responses to interventions. Of particular concern, models with similar parameter sets generated divergent post‐intervention predictions, while models with different parameter sets generated similar ones. This highlights a broader concern: without proper validation, ecosystem models may yield misleading conclusions.

It is reasonable to ask whether our models might have performed better with increased complexity, stronger nonlinearities, or alternative calibration methods. We explored each possibility in our original paper. We tested a regularisation technique that penalised overfitting (as suggested by Woodstock and Harris (Woodstock 2025)), used information‐theoretic model selection, introduced a more nonlinear model variant, and experimented with an alternative fit metric. None improved the performance of Lotka‐Volterra models. While adding density‐dependent compensatory feedback, as suggested by Woodstock and Harris (Woodstock 2025), might reduce some aspects of model misspecification, we remain sceptical that it would resolve the core issues. Such modification does not address parameter non‐identifiability, the curse of dimensionality, or numerical instabilities (Clarke 2008; Evangelou 2022; Wieland 2021)—issues that are often worsened by, rather than alleviated through, increased model complexity.

It is also reasonable to wonder if the problems we found were caused by data rather than a model limitation. Our original analysis was based on data from controlled microcosm experiments. Therefore, observation and process noise were minimised, and importantly, the system was fully observable—all species were accounted for. Given available data sets are typically shorter and noisier, and ecosystems often only partially observed (which increases the likelihood of structural non‐identifiability in models (Wieland 2021)), we believe that the problems we encountered are likely to be even more severe in real‐world scenarios. Nevertheless, it is also plausible that the difficulties we encountered with prediction and inference arose from model misspecification—that is, even with ideal data, the model may not have adequately captured the underlying ecosystem dynamics.

Ecosystem modelling is a central tool in ecology and conservation. However, the technical and data limitations we found are not unique to Lotka‐Volterra models; rather, they are likely pervasive across many ecosystem modelling approaches. Thus, our results might point out to broader issues in ecological modelling—particularly in generating reliable predictions. The first step toward progress is radical honesty about current model performance. We are beginning to see encouraging moves in this direction, particularly within marine ecosystem modelling, through calls for quantitative validation and explicit treatments of uncertainty (Olsen 2016). While such practices are not yet widespread (Stow 2009; Steenbeek 2021) and often reveal suboptimal model performance (Frank 2014; Craig 2023), they represent an important foundation for building more reliable models.

Our results show that a widely used model performs poorly even in a simple, well‐observed system, with data far better than typically available. Neither added complexity, different selection techniques, nor alternative fitting procedures improved performance, underscoring the urgent need for a more critical and systematic approach. Overlooking these model inadequacies may hinder the field's development and undermine trust in mathematical ecology. To advance ecosystem modelling, attention must be given not only to model development, but also to rigorous validation.

## Author Contributions

All authors conceptualised the article. L.L.B. prepared the initial draft, and all authors reviewed and approved the final manuscript.

## Data Availability

The authors have nothing to report.
